# Pharmacokinetic Comparison of Ferulic Acid in Normal and Blood Deficiency Rats after Oral Administration of *Angelica sinensis*, *Ligusticum chuanxiong* and Their Combination

**DOI:** 10.3390/ijms13033583

**Published:** 2012-03-16

**Authors:** Weixia Li, Jianming Guo, Yuping Tang, Huan Wang, Meiyan Huang, Dawei Qian, Jin-Ao Duan

**Affiliations:** Jiangsu Key Laboratory for High Technology Research of TCM Formulae, Nanjing University of Chinese Medicine, Nanjing 210046, China; E-Mails: liweixia01@126.com (W.L.); njuguo@njutcm.edu.cn (J.G.); huanmao1985@163.com (H.W.); maomilove321@163.com (M.H.); qiandw@njutcm.edu.cn (D.Q.)

**Keywords:** ferulic acid, herb pair, pharmacokinetic, synergic action

## Abstract

Radix Angelica Sinensis (RAS) and Rhizome Ligusticum (RLC) combination is a popular herb pair commonly used in clinics for treatment of blood deficiency syndrome in China. The aim of this study is to compare the pharmacokinetic properties of ferulic acid (FA), a main bioactive constituent in both RAS and RLC, between normal and blood deficiency syndrome animals, and to investigate the influence of compatibility of RAS and RLC on the pharmacokinetic of FA. The blood deficiency rats were induced by injecting 2% Acetyl phenylhydrazine (APH) on the first day, every other day, to a total of five times, at the dosage of 100, 50, 50, 30, 30 mg/kg body mass, respectively. Quantification of FA in rat plasma was achieved by using a simple and rapid HPLC method. Plasma samples were collected at different time points to construct pharmacokinetic profiles by plotting drug concentration *versus* time, and estimate pharmacokinetic parameters. Between normal and blood deficiency model groups, both *AUC*_(0–_*_t_*_)_ and *C*_max_ of FA in blood deficiency rats after RAS-RLC extract administration increased significantly (*P* < 0.05), while clearance (*CL*) decreased significantly. Among three blood deficiency model groups, *t*_1/2α_, *V*_d_, *AUC*_(0–_*_t_*_)_ and *AUC*_(0–∞)_ all increased significantly in the RAS-RLC extract group compared with the RAS group. The results indicated that FA was absorbed better and eliminated slower in blood deficiency rats; RLC could significantly prolong the half-life of distribution, increase the volume of distribution and the absorption amount of FA of RAS in blood deficiency rats, which may be due to the synergic action when RAS and RLC were used together to treat blood deficiency syndrome.

## 1. Introduction

*Angelica sinensis* (Oliv.) Diels (Umbelliferae) is a well known medicinal plant and also a famous traditional Chinese herb widely distributed in western China. The radix of *Angelica sinensis* (RAS), called Danggui, was first recorded in “*Shen Nong Ben Cao Jing*” in the Han Dynasty (200–300 A.D.) [1]. It has been used as one of the most important traditional Chinese medicines in Asia to enrich blood and invigorate blood circulation [2]. Danggui has been regarded as “female’s ginseng” as it was extensively applied to the treatment of gynecological disorders, such as menstrual disorders, amenorrhea, dysmenorrhea, and premenstrual syndrome [3–5]. Chuanxiong, the rhizome of *Ligusticum chuanxiong* Hort (RLC) (Umbelliferae), another well known traditional Chinese herb, has been widely used to treat cardiovascular disorders such as stroke, hypertension, and arrhythmia [6]. Moreover, RLC has been prescribed for “dispersal of tissue stasis, removal of chronic inflammation and facilitation of tissue perfusion” [6]. From data mining in a Chinese medicine formula database, the simultaneous usage of two herbs (RAS and RLC) appears over 1200 times [7] and thus it is considered as a herb pair which is a basic unit in formulae, as well as an important bridge between single herb and formulae according to Traditional Chinese Medicine (TCM) theory. When two single herbs were applied as herb pair they may produce a synergistic, an additive, or an antagonistic effect. Interestingly, these two herbs can be used together as unique formulae named Fo-Shou-San (FSS), which contains RAS and RLC in a weight ratio of 3:2. FSS was prescribed for the treatment of women’s ailments, especially obstetric diseases, including dystocia, vaginal bleeding with fetal movement, dead fetus in uterus, and postpartum anemic fainting [8,9]. On the basis of the herb pair, many classical formulae were composed under the guidance of TCM theory, such as Si-Wu-Tang (RAS, RLC, Radix Rehmanniae, and Radix Paeonia in a ratio of 1:1:1:1) [10,11] and its serial formulae [12,13].

RAS and RLC are both famous Chinese herbs from the Umbelliferae plants. They contain many aromatic acids and phthalides as major bioactive constituents. The aromatic acids, including among others ferulic acid, chlorogenic acid, caffeic acid, protocatechuic acid, vanillic acid, have antioxidant, antimicrobial, anti-inflammatory, and anti-thrombosis activities [14–16]. The phthalides, including ligustilide, butylidenephthalide, senkyunolide I, senkyunolide H, have antifungal, antibacterial, anti-inflammatory and antioxidant activities, as well as smooth muscle relaxant, and vasodilation activities [4,10,17]. Ferulic acid (FA), a characteristic aromatic acid in both RAS and RLC [18], is usually used as one of the forming compounds to assess quality [19] and has been clinically used to treat angina pectoris and hypertensive diseases in China [20]. Previous investigations suggested that it could significantly improve blood fluidity, inhibit platelet aggregation, decrease serum lipids, prevent thrombus formation, protect neuron like PC12 cells, and exhibit strong antioxidant activity [17,18,21–24]. FA was also reported to have anti-inflammatory action [25], can prevent ethanol-induced liver injury [26], contribute to the defense against viral infections including AIDS [27], as well as suppress the production of interleukin-8 (IL-8) which was the main cause of the local accumulation of neutrophils, and modulate various inflammatory reactions [25]. Since FA has been demonstrated to exhibit so many pharmacological effects, the understanding of pharmacokinetics of FA is useful for designing and dosing regimens in pharmacological studies. Furthermore, the pharmacokinetic profile can contribute to the safety and efficacy of FA in clinical applications.

The pharmacokinetics of FA *per se* and/or in the TCM extracts [28] and combined formulae [29,30] have been studied in several works, and all performed in normal animals. However, little work has been carried out to investigate pharmacokinetics of FA in pathological state. It is well known that the pharmacokinetic process of a drug may be altered when the body is in morbidity, even significantly different from that in normal conditions. Therefore, it is necessary to study the potential alterations of pharmacokinetic parameters under pathological conditions. Furthermore, the data obtained from pathological conditions could be more beneficial than those from normal conditions in clinical applications.

RAS and RLC, especially the herb pair RAS-RLC, were clinically used for treatment of women’s ailments resulting from blood deficiency in TCM. In the herbs, FA was found to exhibit significant effects on syndrome of blood deficiency [31]. Therefore, in this study, the pharmacokinetic profile of FA was investigated and compared in normal and blood deficiency rat plasma after oral administration of RAS, RLC, and RAS-RLC. Moreover, the related pharmacokinetic profiles of FA are evaluated to analyze the different pharmacokinetic processes in normal and pathological conditions, and to discuss the variation after RAS and RLC were combined into herb pair.

## 2. Results

### 2.1. General and Hemogram Observation of Rats

Normal rats are strong, vigorous, with brilliant pink eyes, clean pink moist nose and lips, round pink tail, straight back and loin, their fur is luxuriant and lustrous. Three days after APH administration, the animals appeared exhausted, sluggish, body curled into a ball with raised hairs, asthmatic and somnolent, tail, face, ears and eyes pale and cool. The signs of blood deficiency rats matched the same description of blood deficiency symptoms.

Additionally, the periphery blood indexes in normal and blood deficiency rats are shown in Table 1, including white blood cell (WBC), red blood cell (RBC), hemoglobin (HGB), and hematocrit (HCT) which were the main diagnostic criteria of blood deficiency syndrome. Results showed that RBC, HGB, and HCT in blood deficiency rats reduced significantly (*P* < 0.05), while WBC increased significantly (*P* < 0.05) in comparison with normal rats, indicating that the blood deficiency model was successfully induced [32].

### 2.2. HPLC Chromatograms

**Figure 1:**
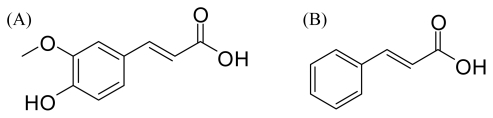
Chemical structures of: (**A**) ferulic acid (FA); and (**B**) cinnamic acid (IS).

**Figure 2:**
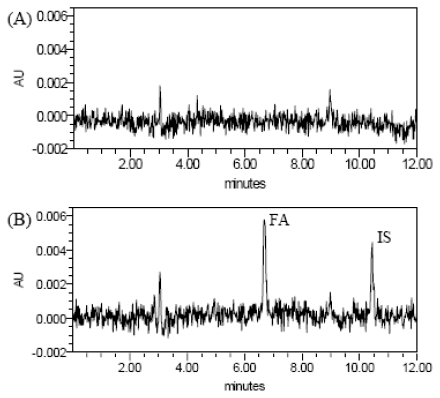

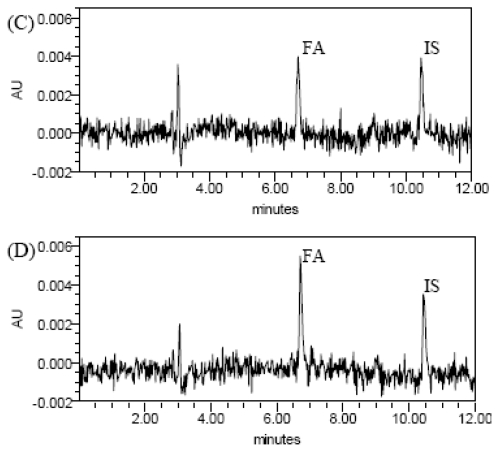
Representative chromatograms of blank plasma sample (**A**), blank plasma sample spiked with FA at 1.095 μg/mL and IS (**B**), plasma sample of normal rats at 15 min after oral administration of Radix Angelica Sinensis (RAS)-Rhizome Ligusticum (RLC) extract (**C**) and plasma sample of blood deficiency rats at 15 min after oral administration of RAS-RLC extract (**D**).

Under the conditions described above, the typical HPLC chromatograms of blank plasma, blank plasma spiked with FA and IS (IS: cinnamic acid; the chemical structures of FA and IS were given in Figure 1), and plasma samples, obtained 15 min after administration of RAS-RLC extract, from normal and blood deficiency rats are presented in Figure 2. The retention times of FA and IS were approximately 6.7 and 10.4 min, respectively. No significant endogenous peaks were observed within the time frame in which FA and IS were detected.

### 2.3. Validation of Chromatographic Methods

The peak area ratio of FA to IS *vs*. the FA concentration curve was linear over the range of 0.1095~10.95 μg/mL, and the detection limit was 0.10 μg/mL. With the least-squares method, a regression equation of *C* (μg/mL) = 8.5265*A*_FA_/*A*_IS_ − 0.5074 (*r* = 0.999 7), where *A*_FA_/*A*_IS_ was the peak area ratio of ferulic acid to cinnamic acid, and *C* was the ferulic acid concentration in μg/mL was obtained. The coefficients of variation (RSD) values of intra-day assay were 1.22, 3.67 and 4.85% at high, medium, and low concentrations of FA, respectively (*n* = 5). The coefficients of variation (RSD) values of inter-day assay were 2.85, 4.00 and 8.77% at high, medium, and low concentrations of FA, respectively (*n* = 5). The accuracy of FA was within the range of 81.12~98.98%. The recovery rates of FA from rat plasma were 88.03 ± 2.98%, 85.54 ± 1.75% and 82.25 ± 2.03% at high, medium, and low concentrations of FA, respectively. The coefficients of variation (RSD) of recovery rates were less than 10%. The results of precision and recovery rates were conformed to the principle of bio-sample analysis.

### 2.4. Determination of FA in Plasma and Pharmacokinetic Analysis

**Figure 3:**
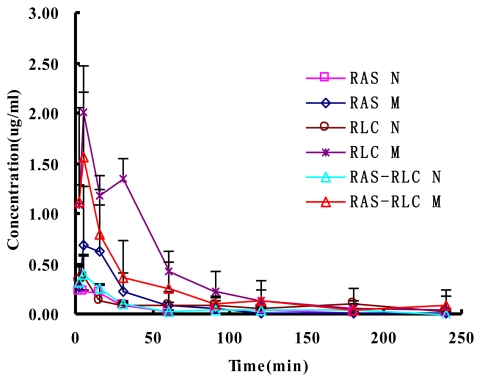
Plasma concentration-time curves of FA in normal (N) and blood deficiency rats (M) after oral administration of RAS, RLC and RAS-RLC extract (at a dose of 6.48, 7.19, 7.35 mg/kg FA). Each point and bar represents the mean ± S.D. (*n* = 6).

The validated method was successfully applied to the pharmacokinetic study of FA in rat plasma after oral administration of RAS, RLC, and RAS-RLC extract. The mean concentration-time curves are shown in Figure 3, and the pharmacokinetic parameters are shown in Table 2.

## 3. Discussion

Acetyl phenylhydrazine is a strong oxidant, which has a slowly progressive and oxidative damage effect on RBC, especially interfering with glucose-6-phosphate dehydrogenase in RBC, promoting the changes from hemoglobin to Heinz-body, and making the RBC disintegrate easily, which finally results in hemolytic anemia of the body. The number of RBC then decreases and the content of HGB reduces significantly. As the number of WBC and rRBC pathologically increase, the liver and spleen swells and their color changes to dark red. All the results conform to the essential features of blood deficiency syndrome. It has been a standard procedure to induce rat blood deficiency and it is recorded in several textbooks [33,34] and literatures [35,36]. According to the procedures recorded in these reports and with slight modification, we have successfully reproduced an APH induced blood deficiency model. In order to decrease bleeding, we supplemented physiological saline (1 mL) once every hour by intraperitoneal injection during the period of blood collection. All the experiments of control groups and model groups were operated in a similar manner.

Diagnosis and treatment based on an overall analysis of the illness and the patient’s condition is the essence of TCM theory. Rational drug therapy is dependent upon a basic understanding of the way patients handle drugs (pharmacokinetics) and their response to specific drug effects (pharmacodynamics). Many researches have demonstrated that disease condition will cause the alterations of pharmacokinetic parameters [37–39]. Moreover, traditional Chinese syndrome state and the drugs combination in traditional Chinese recipe could significantly influence the blood drug concentration and their pharmacokinetic parameters after oral administration [40,41]. Until now, there are no pharmacokinetic data concerning FA in TCM extract or of combined formulae in the pathological condition of blood deficiency, which would assist in providing dosing information and thus enhance the safety and efficacy of TCM utilizing FA in clinical applications. Moreover, since TCMs are administered in abnormal conditions in clinical practice, these related results obtained from normal conditions would not prove the clinical efficacy of TCM containing FA.

In the present study, the pharmacokinetic profiles of FA were evaluated and compared after oral administration of RAS, RLC, and RAS-RLC extract. Results showed that the pharmacokinetic parameters of FA after oral administration of RAS-RLC extract were significantly different between normal and blood deficiency rats. The plasma drug concentration-time data of FA in normal and blood deficiency rats were best fitted to a two-compartment open model, coinciding with the results of other researchers [42]. After oral administration of RAS, RLC, and RAS-RLC extracts in model rats of blood deficiency and their respective control rats, the absorption of FA was rapid; FA was detected in plasma from the first blood sampling time (2 min) and the plasma concentration reached its peak (*C*_max_) at 5 min (*t*_max_) for all groups of rats (Figure 3), which was similar to the results found in the literature [42–44]. After reaching a *C*_max_, the plasma concentrations of FA declined in a polyexponential fashion for each group of rats except the RLC M group, in which the plasma concentration of FA at 30 min was higher than that at 15 min, while lower than that at 5 min. which was the phenomenon of two-peaks in pharmacokinetic, mainly caused by the intestinal reabsorption of enterohepatic cycling. At the same time point, the plasma concentrations of FA in model rats were higher than their respective control rats. It was reported that, the pharmacokinetic profile of FA was significantly different after administration of pure FA and RLC decoction with the same dose and administration method. *T*_max_ of pure FA and FA in RLC decoction was 10 and 2 min, respectively [43].

The results in Table 2 showed that after administration of RAS or RLC single herb, the pharmacokinetic parameters of FA in blood deficiency rats had no significant differences compared with their respective normal rats. But *AUC*_(0–_*_t_*_)_ and *C*_max_ of FA increased significantly (*P* < 0.05) in blood deficiency rats administrated with RAS-RLC extract compared with normal rats, while *CL* decreased significantly (*P* < 0.05). After oral administration of RAS-RLC extract, the *AUC*_(0–t)_ and *C*_max_ values of FA increased significantly in blood deficiency rats, which is probably due to an increase in absorption of FA from the gastrointestinal tract of the rat models of blood deficiency. As reported, the liver and spleen of blood deficiency rats induced by acetyl phenylhydrazine are swollen and dark red compared with normal rats due to the tissues being closely correlated with the hematological system [9]. The rats with blood deficiency syndrome reveal abnormal hemorheology with sluggish blood circulation, leading to the change of hemorrheology parameters including the increase of whole blood viscosity, plasma viscosity, hematocrit and fibrinogen [36,45]. FA is mainly absorbed in stomach and small intestine; poor blood circulation will prolong retention time in the stomach and small intestine and cause the increase of FA’s absorption. Liver is one of the main metabolic organs, therefore,it is impossible for FA to be metabolized quickly because of the pathological change (e.g., some metabolic enzymes) in the liver. This phenomenon may well be attributable to the biological changes of some enzymes and the biomembrane transfer abilities under pathological conditions [39,41]. These results demonstrated that there was significant influence on the pharmacokinetics of FA when RAS and RLC were used in combination as a herb pair, which may be due to the synergic action between RAS and RLC. All the results indicated that FA was absorbed better and eliminated slower in blood deficiency rats.

Furthermore, the results in Table 2 show that, among the three normal groups, there were no significant differences between RAS and RAS-RLC groups, RLC and RAS-RLC groups; nor was there any significant difference after dose correction. Pharmacokinetic parameters of *t*_1/2α_ and *V*_d_ in RLC group were increased significantly compared with RAS group, while *K*_10_ and *K*_12_ decreased significantly, but the main pharmacokinetic parameters of *AUC* and *C*_max_ showed no significant differences. It merely indicated that FA of RLC in normal rats had wider distribution and lower rate constants of transport between the central and peripheral compartments compared with RAS, which might be attributed to the difference in components between RAS and RLC. Among the three blood deficiency groups, however, there were significant differences only between RAS and RAS-RLC groups, and there were significant differences after dose correction, too. Pharmacokinetic parameters of *t*_1/2α_, *V*_d_, *AUC*_(0–_*_t_*_)_ and *AUC*_(0–∞)_ increased significantly (*P* < 0.05) in RAS-RLC extract group compared with RAS group. It also revealed that RLC could significantly raise some pharmacokinetic parameters of RAS such as *t*_1/2α_, *V*_d_, *AUC*_(0–_*_t_*_)_ and *AUC*_(0–∞)_ when RAS and RLC used together in blood deficiency rats. In other words, RLC could significantly prolong the half-life of distribution, increase the volume of distribution and the absorption of FA of RAS in blood deficiency rats. This was probably caused by newly produced components when RAS and RLC were used in combination, indicating the synergic action between RAS and RLC. However, there were no significant differences between RLC and RAS-RLC groups, indicating that RLC could prolong the half-life and increase the apparent volume of distribution and absorption of FA in blood deficiency rats. These results suggested that RAS could have a better effect on nourishing and tonifying blood efficacy with the assistance of RLC, and reflecting the scientific essence of mutual promotion and assistance of RAS-RLC as a herb pair.

## 4. Experimental Section

### 4.1. Materials and Extraction

The radix of *Angelica sinensis* (Oliv.) Diels (Umbelliferae) was collected at Min County, Gansu Province, China, in June 2009. The rhizome of *Ligusticum chuanxiong* Hort (Umbelliferae) was collected at Pengzhou Sichuan, China, also in June 2009. They were identified by Dr. Hui Yan (Department of Pharmacognosy, Nanjing University of Chinese Medicine, Nanjing, China). The voucher specimen (No. NJUTCM-20080701) was deposited in the Herbarium of Nanjing University of Chinese Medicine.

The dry herb materials of RAS (2 kg) and RLC (2 kg) were crushed to pieces, extracted with boiling water (1:8) for twice, 2 h for each time, filtered through gauze. The residue was refluxed with 95% ethanol once under the same conditions. Then three filtrates were merged and evaporated by rotary evaporation under vacuum at 60 °C, thus RAS and RLC extract samples were obtained, respectively. The concentration of FA was 0.46 mg/g in the extract of RAS and 0.51 mg/g of RLC. A total 2 kg mixed pieces of RAS-RLC (1:1, w/w) were extracted through the same procedure. The concentration of FA in the extract of RAS-RLC was 0.52 mg/g.

### 4.2. Animals

Male and female Sprague-Dawley (SD) rats (180–220 g) were obtained from Shanghai Slac Laboratory Animal Co. Ltd. (Shanghai), and kept in an environmentally controlled breeding room (temperature: 20 ± 2 °C, humidity: 60 ± 5%) for 1 week before the experiments started. The rats were fasted for 12 h with free access to water prior to the experiments. Animal welfare and experimental procedures were strictly in accordance with the *Guide for the Care and Use of Laboratory Animals* (US National Research Council, 1996) and the related ethics regulations of Nanjing University of Chinese Medicine.

### 4.3. Chemicals and Reagents

The reference standard of FA (98%) and internal standard (IS), cinnamic acid (98%), were purchased from the National Institute for the Control of Pharmaceutical and Biological Products (Beijing, China). *N*-Acetyl phenylhydrazine (APH) was purchased from Tianjin Institute of Fine Chemicals retrocession (Tianjin, China). Acetonitrile of HPLC grade was obtained from Tedia (Fairfield, OH, USA). Ethyl acetate, hydrochloric acid and glacial acetic acid were of analytical grade. Ultrapure water was prepared using an EPED super-purification system (Eped, Nanjing, China).

### 4.4. Drug Administration and Collection of Rat Plasma Samples

Experiments were performed on 18 female and 18 male Sprague Dawley (SD) rats randomly divided into six groups. Three normal control groups and three blood deficiency model groups were administrated of RAS, RLC, and herb pair RAS-RLC extract, respectively. The blood deficiency model groups were given hypodermic injection with 2% APH on the 1st day, every other day a time, and total of five times at the dosage of 100, 50, 50, 30, 30 mg/kg body mass, respectively [32,42]. Orbit blood of rat was collected 1.5 h after the last injection to detect red blood cell (RBC), white blood cell (WBC), hemoglobin (HGB), and hematocrit (HCT).

On the 10th day, the animals were given intragastrically RAS, RLC, and RAS-RLC extract dissolved and dispersed homogeneously in ultrapure water. The animal dose of RAS, RLC, and RAS-RLC extract was extrapolated from the human daily dose, using the body surface area normalization method [46]. The dose (8.10 g crude herbs per 1 kg rat body weight) of RAS, RLC, and RAS-RLC extract was equivalent to five times of the adult daily dose herb pair RAS-RLC (18 g, from Si-Wu-Tang in which RAS, RLC, Rehmanniae Radix, and Paeonia Radix were 9 g, respectively) crude herbs based on the traditional Chinese medicine prescription, at a dose of 6.48, 7.19, 7.35 mg/kg FA (1.5 mL extract per 100 g body weight). The formula for dose translation was as follows: human dose of crude herbs in clinic × 0.018/200 × 1000 × the multiple of clinical equivalency dose. Blood samples (0.5 mL) were collected at the designated time points (0, 2, 5, 15, 30, 60, 90, 120, 180, and 240 min) after dosing into heparinized Eppendorf centrifuge tubes. Plasma was prepared by centrifuging each blood sample at 3000× *g* for 10 min and the resulting plasma layers were stored in Eppendorf centrifuge tubes at −20 °C until analysis. Data from these samples were used to construct the pharmacokinetic profiles by plotting drug concentration *vs.* time curves.

### 4.5. Plasma Sample Preparation

The thawed plasma samples (100 μL) were transferred to a 1.5 mL Eppendorf centrifuge tube, added with 20 μL IS (0.37 mg/mL) and then mixed with 50 μL 0.1 mol/L hydrochloric acid and 850 μL ethyl acetate. Afterwards mixture was vortexed for 1 min and centrifuged at 3000× *g* for 5 min at ambient temperature. Supernatant (800 μL) was transferred into a 1.5 mL Eppendorf centrifuge tube and evaporated to dryness under the stream of nitrogen at 40 °C, the residue was dissolved in 100 μL of mobile phase, and the mixture was vortexed for 1 min and centrifuged at 13,000× *g* for 5 min at ambient temperature. Finally, a 10 μL aliquot of supernatant was injected into HPLC for analysis.

### 4.6. Chromatographic Conditions

Analysis was performed on a Waters 2695 Alliance HPLC system (Waters Corp., USA) equipped with a 2996 photodiode array detector (PDA) together with a quaternary pump solvent management system, an on-line degasser and an autosampler. The analytes were separated on a Hedera™ ODS-2 column (250 mm × 4.6 mm, 5 μm) from Jiangsu Hanbon Sci and Tech Co. Ltd. (Jiangsu, China). The mobile phase was composed of solvent A (0.3% aqueous glacial acetic acid, *v*/*v*) and B (acetonitrile), the gradient program was set as follows: 20–40% B at 0–5 min, 40–60% B at 5–12 min. The flow rate was 1.0 mL/min and detection wavelength was set at 320 nm. The operating temperature was maintained at 35 °C.

#### 4.6.1. Linearity

Primary stock solutions of FA and IS were prepared by dissolving accurately weighed FA and IS in methanol to yield a final concentration of 0.219 and 0.37 mg/mL, respectively. For the calibration curve, six concentrations (0.1095, 0.5475, 1.095, 2.19, 4.38, 10.95 μg/mL) of FA solution were prepared by dilution of the stock solution. The serial solutions (10 μL) were spiked in 100 μL blank plasma, then 20 μL of the IS solution were added. Afterwards, calibration standards were processed and assayed as described above, and 10 γL aliquot of supernatant was injected into HPLC for analysis. The calibration curves in the range of 0.1095~10.95 μg/mL for FA were constructed by plotting the ratio of peak area between FA and IS against the FA standard concentrations using least-square method.

#### 4.6.2. Recovery and Accuracy

Three concentrations (high, medium, and low) of standard solution mixture in the blank rat plasma were detected five times on the same day for intra-day and continuously for five days for the inter-day accuracy variation test. The precision coefficient of variation (RSD) was calculated from the observed concentration (*C*_obs_) as following equation: %RSD = [standard deviation (S.D.)/*C*_obs_] × 100. The recovery rates (%) were calculated from the mean value of the observed concentration (*C*_obs_) and the theoretical concentrations (*C*_the_) as following equation: % = [*C*_obs_/*C*_the_] × 100.

### 4.7. Pharmacokinetic Analysis

The pharmacokinetic data analysis was processed by a pharmacokinetic software package of DAS 2.1 which is edited by the Mathematics Pharmacological Committee, Chinese Pharmacological Society. The values of the experimental data and the pharmacokinetic parameters were expressed as mean γ S.D. The following pharmacokinetic parameters such as elimination half-life (*t*_1/2α_, *t*_1/2β_), area under the plasma concentration-time curve from zero to the last experimental point 240 min (*AUC*_0–240_), the area under the plasma concentration-time curve from zero to infinity (*AUC*_0–limit_), clearance (*CL*), volume of distribution (*V*_d_), rate constants of transport between the central and peripheral compartments (*K*_12_ and *K*_21_), the elimination rate constant (*K*_10_), the maximum concentration (*C*_max_), and time of maximum plasma concentration (*t*_max_) were calculated by the compartmental pharmacokinetic analysis. A statistical analysis was performed using an analysis of variance (ANOVA). A *P* value < 0.05 was considered statistically significant and *P* < 0.01 being very significant.

## 5. Conclusion

This study suggested that the pharmacokinetic process of herbs will alter in pathological conditions, and can be influenced by other herbs when they are used as a herb pair or formulae. The knowledge of the pharmacokinetic processes of herb pair or formulae in normal and pathological conditions can help us explain and predict a variety of events related to the efficacy and toxicity of TCM.

## Figures and Tables

**Table 1:** The periphery blood index in normal and blood deficiency rats (χ̄ ± *s*).

Group	WBC/10^9^·L^−1^	RBC/10^12^·L^−1^	HGB/g·L^−1^	HCT/%
Normal	14.78 ± 3.31	7.01 ± 1.59	140.87 ± 10.22	0.42 ± 0.04
Model	133.25 ± 10.04 ^**^	3.72 ± 0.88 ^**^	92.33 ± 5.28 ^**^	0.33 ± 0.05 ^**^

Compared with normal group: ^**^ *P* < 0.01. White blood cell (WBC), red blood cell (RBC), hemoglobin (HGB), and hematocrit (HCT).

**Table 2:** Pharmacokinetic parameters of FA in normal and blood deficiency rats plasma after oral administration of RAS, RLC and RAS-RLC extract (at a dose of 6.48, 7.19, 7.35 mg/kg FA).

Parameters	Normal			Blood deficiency		
	
	RAS	RLC	RAS-RLC	RAS	RLC	RAS-RLC
	
*t*_1/2α_ (min)	3.902 ± 0.783	28.728 ± 5.813 ^Δ^	28.797 ± 5.208	5.342 ± 0.802	31.231 ± 3.493	28.984 ± 3.066 ^#^
*t_1_*_/2β_ (min)	45.785 ± 6.224	53.125 ± 7.663	37.126 ± 2.545	21.787 ± 2.693	23.121 ± 4.005	30.117 ± 2.056
*V*_d_ (L/mg)	7.965 ± 1.002	50.882 ± 8.135 ^ΔΔ^	23.005 ± 3.031	4.318 ± 0.228	9.633 ± 2.209	14.972 ± 0.206 ^#^
*CL* (L/min/kg)	0.886 ± 0.237	0.905 ± 0.218	0.760 ± 0.186	0.732 ± 0.056	0.327 ± 0.093	0.114 ± 0.002 ^*^
*AUC*_(0–_*_t_*_)_ (mg/L·min)	21.649 ± 5.292	16.925 ± 3.368	20.194 ± 5.000	38.504 ± 4.912	88.685 ± 5.589	118.336 ± 5.876 ^* ##^
*AUC*_(0–∞)_ (mg/L·min)	25.689 ± 2.664	29.013 ± 2.276	27.412 ± 3.638	51.487 ± 4.240	94.457 ± 5.898	175.970 ± 3.437 ^##^
*K*_10_ (1/min)	0.759 ± 0.018	0.018 ± 0.004 ^Δ^	0.035 ± 0.015	0.309 ± 0.043	0.077 ± 0.000	0.008 ± 0.000
*K*_12_ (1/min)	0.615 ± 0.187	0.002 ± 0.000 ^ΔΔ^	0.043 ± 0.003	0.318 ± 0.059	0.030 ± 0.000	0.013 ± 0.001
*K*_21_ (1/min)	0.040 ± 0.015	0.022 ± 0.001	0.013 ± 0.003	0.070 ± 0.024	0.012 ± 0.000	0.014 ± 0.001
*T*_max_ (min)	9.898 ± 1.025	5.000 ± 0.000	8.775 ± 1.202	9.996 ± 0.889	16.667 ± 2.583	15.000 ± 0.010
*C*_max_ (mg/L)	0.444 ± 0.112	0.321 ± 0.026	0.582 ± 0.124	0.918 ± 0.320	1.962 ± 0.042	1.088 ± 0.202 ^*^

Blood deficiency groups compared with their normal groups, respectively (^*^ *P* < 0.05); Compared with normal group (RAS) (^Δ^ *P* < 0.05, ^ΔΔ^ *P* < 0.01); Compared with blood deficiency group (RAS) (^#^ *P* < 0.05, ^##^ *P* < 0.01).
